# Mitochondrial Damage and Mitochondria-Targeted Antioxidant Protection in LPS-Induced Acute Kidney Injury

**DOI:** 10.3390/antiox8060176

**Published:** 2019-06-14

**Authors:** Egor Y. Plotnikov, Irina B. Pevzner, Ljubava D. Zorova, Valery P. Chernikov, Andrey N. Prusov, Igor I. Kireev, Denis N. Silachev, Vladimir P. Skulachev, Dmitry B. Zorov

**Affiliations:** 1 A.N. Belozersky Institute of Physico-Chemical Biology, Lomonosov Moscow State University, Moscow 119992, Russia; irinapevzner@mail.ru (I.B.P.); lju_2003@list.ru (L.D.Z.); panikol@mail.ru (A.N.P.); iikireev@gmail.com (I.I.K.); silachevdn@belozersky.msu.ru (D.N.S.); skulach@belozersky.msu.ru (V.P.S.); 2 V.I. Kulakov National Medical Research Center of Obstetrics, Gynecology and Perinatology, Moscow 117997, Russia; 3 Institute of Molecular Medicine, Sechenov First Moscow State Medical University, Moscow 119991, Russia; 4 Research Institute of Human Morphology, Moscow 117418, Russia; 1200555@mail.ru

**Keywords:** newborn, kidney, sepsis, mitochondria, inflammation, oxidative stress, antioxidants

## Abstract

Induced and frequently unwanted alterations in the mitochondrial structure and functions are a key component of the pathological cascade in many kidney pathologies, including those associated with acute damage. One of the principal pathogenic elements causing mitochondrial dysfunction in Acute Kidney Injury (AKI) is oxidative stress. After ischemia and nephrotoxic action of drugs, sepsis and systemic inflammation are the most frequent causes of AKI. As the kidney suffers from oxidative stress during sepsis, one of the most promising approaches to alleviate such damaging consequences is the use of antioxidants. Considering administration of lipopolysaccharide (LPS) as a model of sepsis, we demonstrate that the mitochondria of neonatal renal tissue are severely affected by LPS-induced AKI, with pathological ultrastructural changes observed in both the mitochondria of the renal tubular epithelium and the vascular endothelium. Upon mitochondrial damage, we evaluated the effect of the mitochondria-targeted antioxidant plastoquinol decylrhodamine 19 (SkQR1) on the development of acute renal failure in newborn rats associated with systemic inflammation induced by the administration of LPS. We found that SkQR1 administration 3 h before LPS led to decreased urinal expression of the AKI marker neutrophil gelatinase-associated lipocalin 2 (NGAL), in addition to a decrease in urea and creatinine levels in the blood. Additionally, an observed impairment of proliferative activity in the neonatal kidney caused by LPS treatment was also prevented by the treatment of rat pups with SkQR1. Thus, one of the key events for renal tissue damage in neonatal sepsis is an alteration in the structure and function of the mitochondria and the mitochondria-targeted antioxidant SkQR1 is an effective nephroprotective agent, which protects the neonatal kidney from sepsis-induced AKI.

## 1. Introduction

Sepsis is a complex syndrome, arising from bacterial invasion and the development of systemic inflammation, which eventually leads to multi-organ failure and, often, to death [1]; even with treatments using antibiotics. The main challenges in the treatment of sepsis and the accompanied dysfunctions of various organs are associated with the lack of clinical and pre-clinical studies of therapeutic approaches which show a positive outcome based on a comprehensive analysis of their effectiveness and safety [2]. Consequently, the research community recently came to the conclusion that the previously proposed approaches in treating sepsis with anti-inflammatory drugs had been proven to be ineffective [3,4].

On the other hand, increasing data has been accumulated which has suggested that the changes in cellular redox homeostasis may play a very important and leading role in the pathogenesis of sepsis and that mitochondria are an essential element in this pathological cascade [5,6,7]. Interestingly, the key role of mitochondria in the development of sepsis and its negative effects on the organism was suggested as early as 1995 [8], with this issue being addressed more systematically after 2001 [9,10].

However, most approaches and studies have, so far, been focused on sepsis in adults, while neonatal sepsis is perhaps a much more critical problem, as the immune system of newborns (especially in pre-term stages) seriously differs from that of the adult organism [11,12]. As a result, a number of immune reactions, such as the response of neutrophils to bacterial invasion and systemic inflammatory reactions—including those that arise during sepsis—occur in neonates in a manner that is quite different, in comparison to adults [13].

One of the most vulnerable organs during sepsis is the kidney. Among patients suffering from sepsis, more than 50% develop an acute kidney injury (AKI) and the mortality rate can be as high as 60% [14]. In the case of neonatal sepsis, the kidney is even more susceptible to damage, due to incomplete nephron development, which usually proceeds with active cell division [15]. In adult organisms, renal pathologies, such as ischemia, myoglobinuria, and nephrotoxicity of drugs, are known to be highly associated with oxidative stress [16,17,18], which is a key damaging factor and mostly arises from mitochondrial dysfunction. This factor is also greatly pathogenic in sepsis, with many researchers being inclined to consider that one solution to the problem of sepsis and the accompanying multiple organ failure may lie with the normalization of redox homeostasis during impaired mitochondrial functions.

As the redox balance is important for the functioning of many cellular systems and as reactive oxygen species (ROS) often play a principal role in the transduction of positive signals [19,20], the use of large doses of conventional untargeted antioxidants is not always a reasonable approach [21]. Therefore, for the treatment of sepsis, the proposed approaches are aimed at extinguishing the mitochondrial-specific oxidative burst, primarily by using mitochondria-targeted antioxidants.

These approaches can be quite universal in their use, particularly for the treatment of neonatal sepsis, since they counteract unwanted activation of the pathogenic redox cycle of ROS production stemming from the mitochondrial respiratory chain, which may be subject to amplification; thus, potentially creating a pathological damaging signal. In this cascade, mitochondria, being a primary source of signaling ROS, can produce ROS in an amount exceeding normal physiological levels, leading to greater mitochondrial dysfunction through a self-amplifying ROS signal, which can have consequences beyond the mitochondrion [22]. All of these processes have been observed for the kidney under conditions of systemic inflammation [23] and, therefore, targeted effects on mitochondria and redox homeostasis can be a very promising approach to reduce the negative impact of neonatal sepsis on the kidney.

As the potential use of mitochondrial antioxidants to protect the kidney has been demonstrated with positive outcomes in a variety of models associated with oxidative stress, we opted using such an approach for AKI analysis in newborns. In this study, considering the administration of lipopolysaccharide (LPS) as a model of sepsis, we induced renal failure by LPS to assess the nephroprotective action of the mitochondria-targeted compound plastoquinone decylrhodamine 19 (SkQR1), which demonstrated remarkable antioxidative and protective properties in a model of renal ischemia and myoglobinuria [24].

## 2. Materials and Methods 

### 2.1. Animal Experiments

Outbred white rats, kept in an animal housing facility, were used with a 12-hour light cycle at a constant temperature (of 22 ± 2 °C). The experiments were conducted in accordance with the ethical standards and recommendations for accommodation and care of laboratory animals, covered by the Council Directives of the European community 2010/63/EU on the use of animals for experimental studies. The animal protocols were approved by the institutional animal ethics committee (Protocol 5/18 from May 14, 2018). The number of pups in a litter was 9–12 and the experiments were conducted on 7-day-old pups of both genders, weighing 9 to 14 g.

### 2.2. Modeling Septic Kidney Damage

Rat pups from the same litter were randomly divided into three groups: (1) Control, intact animals; (2) LPS, rats that received an intraperitoneally (i/p) injection of LPS of *E. coli* strain 0127:B8 (Sigma Aldrich, St. Louis, MO, USA) at a dose of 4 mg/kg; and (3) LPS + SkQR1, rats that were treated with the mitochondria-targeted antioxidant SkQR1, administered i/p at a single dose of 100 nmol/kg 3 h prior to LPS treatment. The SkQR1 was synthesized at the A.N. Belozersky Institute, Moscow State University. The protocol of SkQR1 administration was chosen based on our earlier studies, where the most effective concentration and timing were selected. The total number of animals in each group was 12 (from different litters). After exposure, the animals were returned to their mothers and, after 24 h, samples of blood, urine, and kidneys were taken for Western blotting analysis. Urine samples obtained from rats by bladder puncture were centrifuged at 10,000× *g* and mixed with sample buffer containing 10% 2-mercaptoethanol. Samples were boiled for 5 min and 20 μL of the sample was placed into the well of the gel. Blood urea nitrogen (BUN) and serum creatinine were determined using the AU480 Chemistry System (Beckman Coulter, Brea, CA, USA).

### 2.3. Western Blotting

For the analysis of kidney damage, the markers kidney injury molecule-1 (KIM-1) and neutrophil gelatinase-associated lipocalin 2 (NGAL) were measured in urine collected from pups 24 h after treatment. For sampling, the urine was diluted 4-fold in sample buffer containing 10% 2-mercaptoethanol. Samples were boiled for 5 min and 20 μL of the sample were analyzed by sodium dodecyl sulfate–polyacrylamide gel electrophoresis (SDS–PAGE).

Proliferative cell nuclear antigen (PCNA) was quantified in kidney homogenates as a marker for cell proliferation. Animals were sacrificed by decapitation, after which the kidneys were removed and quickly chilled in ice-cold phosphate-buffered saline (PBS). The kidney was dissected into fragments and then homogenized in 0.5 mL of PBS, containing 1 mM of protease inhibitor phenylmethylsulfonyl fluoride (PMSF). The resulting homogenate was centrifuged at 3000 rpm for 3 min, the supernatant was mixed with 4x sample buffer containing 10% 2-mercaptoethanol, and boiled for 5 min. An aliquot of the supernatant was used to determine the concentration of total protein using a commercial kit based on bicinchoninic acid (Sigma Aldrich, St. Louis, MO, USA). Equal amounts of protein were analyzed by SDS–PAGE. Separated proteins were transferred onto polyvinylidene difluoride (PVDF) membranes (Amersham Pharmacia Biotech, Rainham, UK) by semi-dry blotting. Membranes were blocked for 1 h at 25 °C in PBS with 5% non-fat dry milk and 0.05% Tween-20, incubated with primary antibodies against NGAL, KIM-1 (Abcam, Cambridge, UK), and PCNA (Abcam, UK) at a dilution of 1:1000 in PBS/BSA/Tween-20 and, then, with secondary antibodies conjugated with horseradish peroxidase at a dilution of 1:10,000 in PBS/Tween-20. Bands were detected using a chemiluminescent substrate for horseradish peroxidase ECL (Enhanced chemiluminescence system, Amersham Pharmacia Biotech, Amersham, UK). Chemiluminescence was detected with the ChemiDoc instrument MP Imaging System (Bio-Rad, Hercules, CA, USA) and the obtained images were analyzed using the ImageLab program.

### 2.4. Transmission Electron Microscopy

The cortical area of the kidney was cut into pieces approximately 1 mm in size and fixed with 2.5% glutaraldehyde (Sigma, USA) on the Sorensen phosphate buffer (pH 7.4). Fixed samples were stained with 1% OsO_4_ in phosphate-buffered saline (PBS), followed by dehydration in ascending acetone concentrations, stained with 1% uranyl acetate in 70% acetone during dehydration and embedded in EPON™–Araldite mixture resin. After polymerization, 80 nm thick sections were made using the LKB Nova ultramicrotome (Stockholm, Sweden). Sections were collected on a carbon-coated Cu grid, stained with lead citrate, according to Reynolds (1963), and viewed with the electron microscope JEM-1400 (“JEOL”, Tokyo, Japan) at a 100 kV accelerating voltage.

### 2.5. Statistics

All data are presented as mean ± standard error of the mean (SEM). Comparisons between groups were made using a Student t-test and with a Mann–Whitney test, with a *p* value less than 0.05 taken to have statistical significance.

## 3. Results

### 3.1. Change in the Ultrastructure of Mitochondria in LPS-Induced AKI

**Figure 1:**
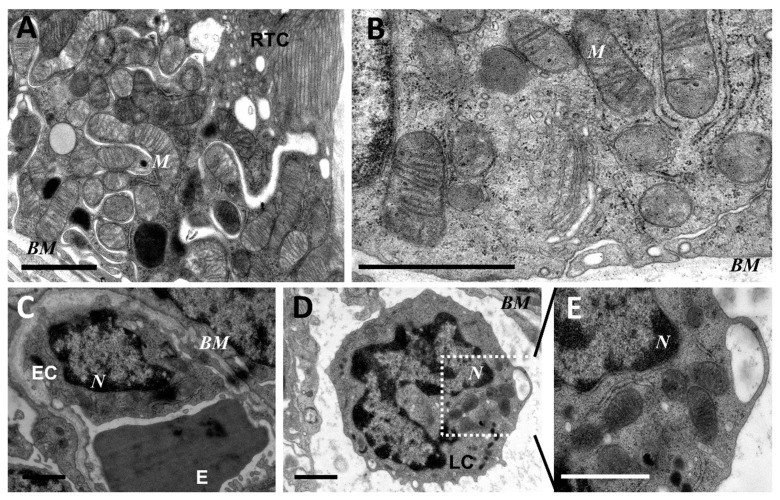
Ultrastructure of mitochondria of renal tubular cells (**A**,**B**), endothelium (**C**), and leukocytes (**D**,**E**) in the intact neonatal kidney. Renal tubular cells (RTC), endothelial cells (EC), and leukocytes (LC) contain mitochondria (M) in the orthodox configuration; BM, basal membrane; N, nucleus; E, erythrocyte in the vessel. Scale bar, 1 μm.

**Figure 2:**
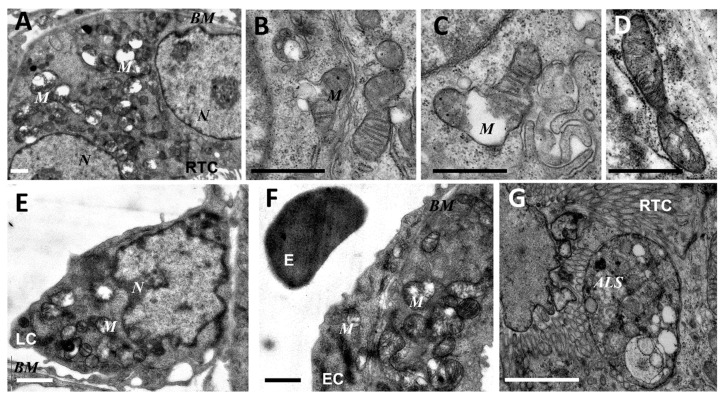
Ultrastructural changes in mitochondria of renal tubular cells (**A**–**D**,**G**), endothelium (**F**), and leukocytes (**E**) after lipopolysaccharide (LPS) administration. Renal tubular cells (RTC), endothelial cells (EC), and leukocytes (LC) contain mitochondria (M) with damaged structures. Alterations in mitochondrial morphology, ultimately, are expressed by extensive swelling of inter-cristae space and matrix. BM, basal membrane; N, nucleus; ALS, autophagolysosome; E, erythrocyte in the vessel. Scale bar, 1 μm.

**Figure 3:**
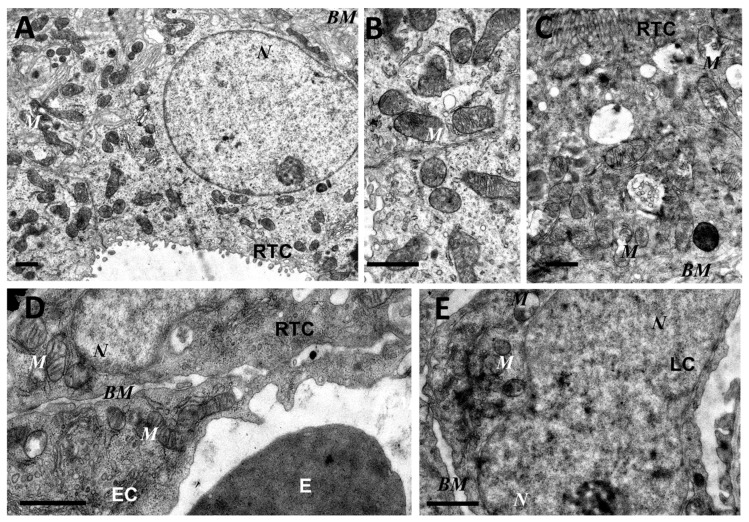
Partial restoration of mitochondrial ultrastructure of renal tubular cells (**A**–**C**), endothelium (**D**), and leukocytes (**E**) after LPS administration with plastoquinol decylrhodamine (SkQR1) treatment. Renal tubular cells (RTC), endothelial cells (EC), and leukocytes (LC) contain a major portion of normal mitochondria (M) in orthodox configuration and few swollen mitochondria (C). BM, basal membrane; N, nucleus; E, erythrocyte in the vessel. Scale bar, 1 μm.

As mitochondria play a decisive role in determining the fate of a cell and the development of oxidative stress under different challenges (including sepsis), we evaluated the effect of LPS treatment on the ultrastructure of the mitochondrial reticulum of renal tubular epithelial cells, as well as endothelial cells in the microvessels of the renal cortex. Electron microscopic examination of the renal cortex revealed significantly impaired mitochondrial ultrastructures in renal epithelial cells 24 h after administration of LPS. Namely, in the tubules of the kidney cortex of the intact kidney, we mainly observed tubular epithelial cells with normal mitochondria maintained in the orthodox conformation (Figure 1A,B). In similar areas of the kidney, fixed 24 hours after the administration of LPS, there were many tubules with altered mitochondrial morphology, in addition to normal epithelial cells containing mitochondria with a preserved classical ultrastructure. In particular, there was a significant enlargement in the inter-crystae and inter-membrane space, accompanied by local enlightenment of the mitochondrial matrix and total mitochondrial swelling (Figure 2A–D). In some mitochondria, there was significant protrusion of the outer membrane, correlating with a breach of the cristae structure until their complete disappearance (Figure 2B,C). These morphological changes were accompanied by signs of degradation of components of the cell carrying such mitochondria; namely, in a number of tubular cells. Structures similar to autophagolysosomes were observed, carrying numerous membranous compartments, and which likely represented partially degraded cell content (Figure 2G). In addition, significant pathological alterations in the structure of mitochondria were observed in the endothelial cells of renal vessels. In these cells, 24 h after sepsis induction, mitochondria with a significantly enlightened matrix were observed, with signs of total swelling and degradation of cristae (Figure 2E,F), whereas, in the intact kidney, endothelial cells predominantly maintained mitochondria in the orthodox conformation. Treatment with SkQR1 before LPS administration led to a pronounced restoration of the mitochondrial ultrastructure in the neonatal kidneys. In renal tubular cells, the major portion of mitochondria restored orthodox configuration (Figure 3A,B), but some cells still contained mitochondria with a swollen matrix (Figure 3C). In endothelial cells (Figure 3D) and leukocytes (Figure 3E), the majority of mitochondria were restored to normal structure.

### 3.2. The Effect of SkQR1 on LPS-Induced Acute Kidney Injury

**Figure 4:**
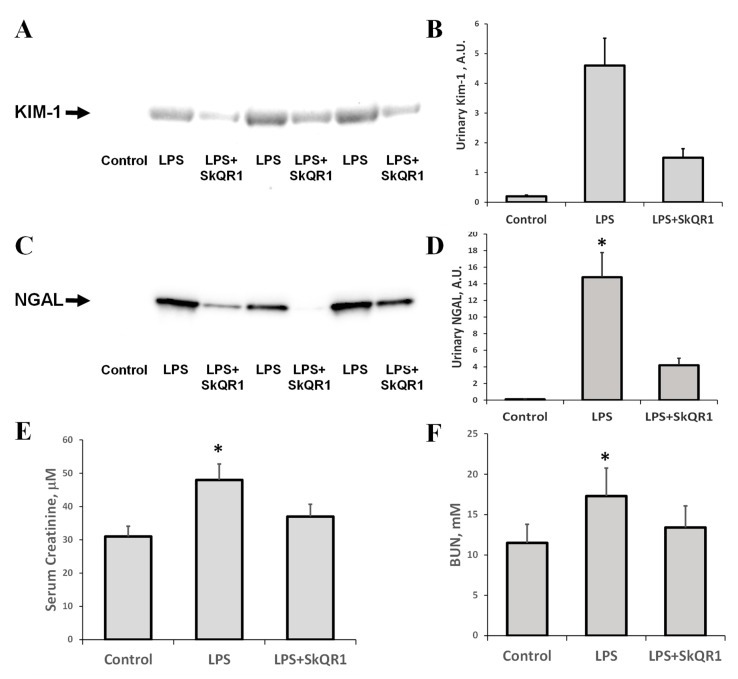
Acute kidney injury (AKI) in neonatal rats 24 h after LPS administration and nephroprotective effect of SkQR1. A significant increase (*, *p* < 0.05) in urinary kidney injury molecule (Kim-1, **A**) and neutrophil gelatinase-associated lipocalin 1 (NGAL, **C**) content were observed, indicating the development of severe AKI. The diagrams in (**B**,**D**) represent densitometry analysis of corresponding bands on the blots. The number of animals for western blot densitometry was *n* = 3 for all groups. SkQR1 treatment protected the kidney from AKI, in terms of KIM-1 and NGAL levels in urine. Renal function was measured as the concentration of creatinine (**E**) and blood urea nitrogen (BUN) (**F**) in blood. A significant (*, *p* < 0.05) increase in serum creatinine and BUN was found after LPS administration; however, SkQR1 treatment restored the levels of these markers.

LPS injection in rats led to acute renal failure, as significant increases in serum creatinine and BUN were observed 24 h after the administration of LPS (Figure 4E,F). Alternative biomarkers of AKI, NGAL, and KIM-1, which were detected in urine after LPS treatment, demonstrated the occurrence of renal damage (Figure 4A–D). Mitochondria-targeted antioxidant SkQR1 administered i/p at a dose of 100 nmol/kg to rats 3 h prior to LPS treatment, in all rats, caused a significant reduction in BUN, creatinine, and urine AKI markers (Figure 4). The KIM-1 and NGAL levels were decreased by 50% in the SkQR1-treated group 24 h after LPS-challenge, compared to untreated neonates. In the kidney tissue of LPS-treated pups, the levels of KIM-1 increased significantly (Supplemental Figure S1A), while the amount of this protein in the normal kidney and that after treatment with SkQR1 was very close to the detection limit. AKI caused by LPS has been associated with the induction of apoptosis, which was manifested by increased caspase-3 levels (Supplemental Figure S1B).

### 3.3. The Effect of SkQR1 on Proliferation in the Kidney during LPS-Induced AKI

**Figure 5:**
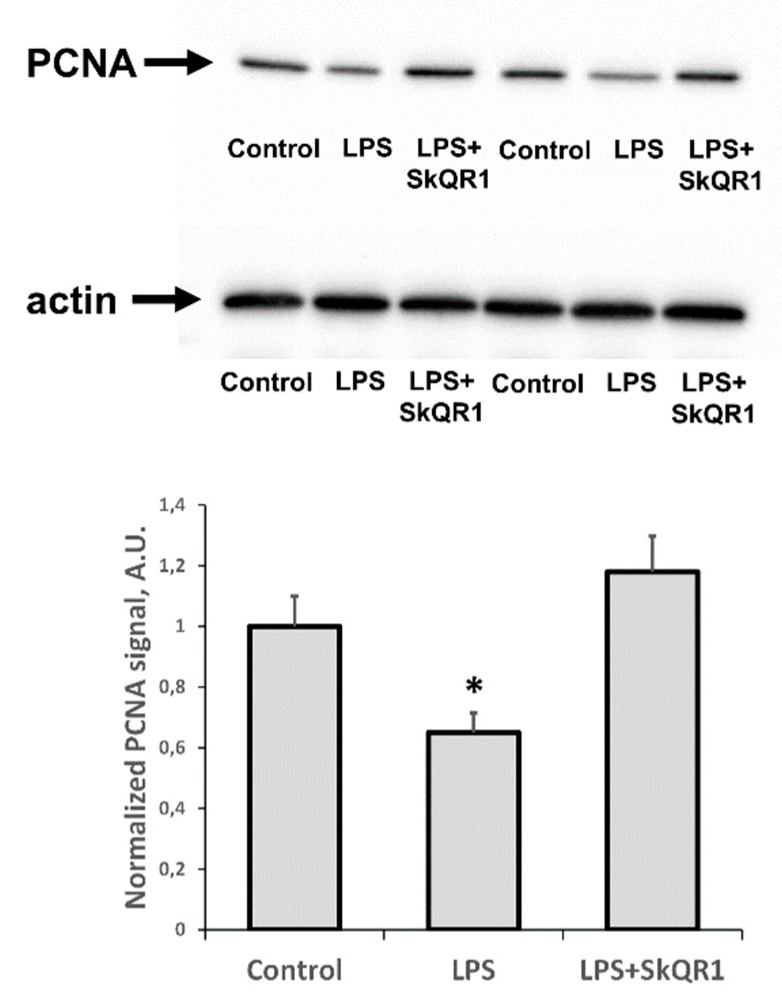
Proliferative activity of kidney cells of neonates 24 h after LPS treatment. Immunoblots for proliferating cell nuclear antigen (PCNA) levels in neonatal kidneys and their densitometry analysis in the diagram, respectively. The SkQR1 treatment restored the levels of PCNA to intact kidney values. Actin was used as a loading control. Number of animals for western blot was *n* = 4. *: *p* < 0.05, compared to control.

In addition to the appearance of AKI markers, we found that LPS caused a significant reduction in the proliferative capacity of renal tissue, as seen by a decrease in PCNA levels (Figure 5). Treatment with SkQR1 greatly restored this proliferative index in the kidney and final renal levels of PCNA after treatment with SkQR1 were similar to control values observed in untreated neonates (Figure 5).

### 3.4. Survival of Animals with LPS-Induced AKI

**Figure 6:**
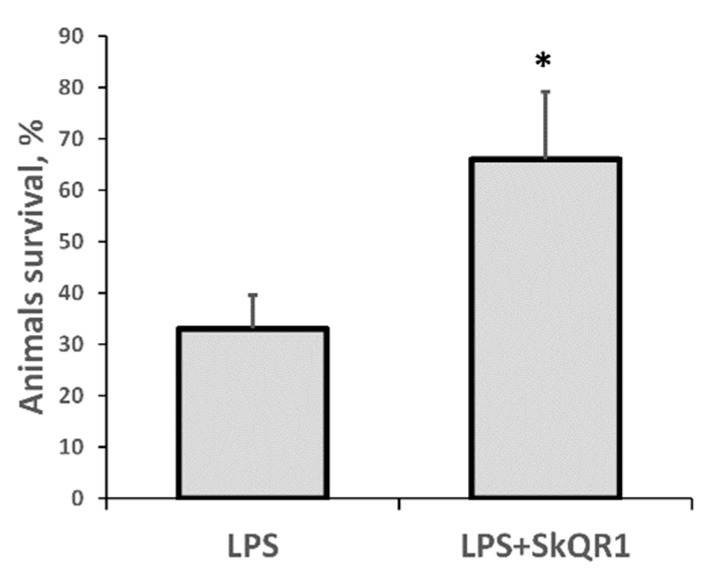
Severe LPS-induced sepsis resulted in high neonatal mortality, which was prevented by SkQR1 treatment. Number of animals in each group was *n* = 6. * *p* < 0.05, compared to LPS alone.

In our model of septic AKI, the mortality of rats on the first day after LPS administration was substantial, making this model highly relevant to the clinical setting. Remarkably, treatment with SkQR1 3 h before LPS treatment resulted in significantly greater animal survival (Figure 6). These results indicate that mitochondrial ROS production plays a crucial role in LPS-associated kidney damage and subsequent sepsis-induced mortality.

## 4. Discussion

The pathological mechanism of sepsis-mediated organ failure—particularly, kidney damage—is known to be governed by oxidative stress [25,26,27,28], due to the development of a ‘sepsis redox cycle’, a cascade of inflammatory and redox events [29]. Interactions between pathogen-associated molecular patterns (PAMPs) and immune cells trigger a detrimental self-maintaining redox loop through NADPH-oxidase activation, cyclooxygenase-2 over-expression, and, ultimately, an increase of H_2_O_2_ levels in the cytosol with further activation of NF-kB [30]. Although there is sufficient evidence to indicate that mitochondrial pathological ROS are associated with the development of oxidative stress in sepsis [31], it remains unknown whether mitochondrial dysfunction is caused by intrinsic oxidative stress as a result of the intra-mitochondrial ROS self-amplification cascade or vice versa: Oxidative stress arising from extrinsic sources leading to damage of the mitochondria, causing further mitochondrial ROS bursts. It should be noted that, regardless of the primary cause of increased ROS production, mitochondria may organize additional pathological mechanisms; namely, “ROS-induced ROS-release” (RIRR) which generates a pathological ROS signal by circuits where the mitochondrial permeability transition plays a significant role [32]. In turn, this leads to the simultaneous collapse of the mitochondrial membrane potential and a transient pathological ROS burst stemming from the electron transfer chain. Generated ROS can be released into cytosol and trigger RIRR in neighboring mitochondria [22]. Using vital sections of the kidney and brain and ROS sensors, including mitochondria-targeted ones, we have previously demonstrated that mitochondria are significant pathogenic sources of ROS in ischemia/reperfusion [33,34]. However, for neonatal sepsis, there has been no direct evidence of mitochondrial structure and functional impairments in the kidney, to date.

In our work, LPS caused significant mitochondrial ultrastructure impairments, not only in the cells of the renal epithelium, but also in the vascular endothelium. Upon electron microscopic analysis, we observed numerous mitochondria with damaged cristae and impaired integrity of both internal and external mitochondrial membranes, caused by LPS administration. Such morphological changes in mitochondria may indicate dysfunctionality associated with the onset of oxidative stress. For instance, it has been shown, under conditions of modeling sepsis in the liver (by LPS injection), that mitochondrial functions were significantly impaired and that this impairment was strongly correlated with the extent of mitochondrial ultrastructural abnormalities [35]. Additionally, oxidative stress has been reported in patients with sepsis, and mitochondrial dysfunction has been suggested as a causative factor in the development of multiple organ failure [36]. Thus, it can be concluded that mitochondria are one of the key organelles involved in the development of pathogenic cascades under septic conditions, acting both as a source and a target for ROS.

The development of oxidative stress may be prevented or reduced, in terms of its pathological consequences, by removing one of its original causes: the production of pathological ROS in the mitochondria. For these purposes, the use of antioxidant molecules which can be targeted for delivery to the mitochondria has been proposed [37,38]. Targeted delivery of antioxidant molecules is based on the principle that the mitochondria have a potential difference on the inner membrane and that the mitochondrial matrix is negatively charged. As a result, substances having a lipophilic permeable cation in their composition can electrophoretically accumulate in the mitochondria [39,40]. Using this principle, various mitochondria-targeted antioxidants have been created, which differ in both the antioxidant moiety and the lipophilic cation. To test the hypothesis of mitochondrial ROS pathological contribution to sepsis-mediated AKI, we used the mitochondrial-targeted antioxidant SkQR1, a conjugate of plastoquinone containing a rhodamine residue, administered 3 h before LPS treatment. In previous studies, we have shown that intraperitoneal administration of SkQR1 resulted in its rapid accumulation in the mitochondria, both in tubules and in glomeruli of adult rats [41], and protected animals from AKI caused by ischemia/reperfusion of the kidney [24]. We have also shown that the protective effects of SkQR1 can be mediated both by the direct antioxidative properties of this molecule [40,41] and by an indirect reduction in the ROS levels, due to SkQR1-induced mild uncoupling of mitochondria [42]. Additional indirect effects of SkQR1 include induction of cytoprotective signaling pathways, such as synthesis of erythropoietin in the kidney and increase of phosphorylated glycogen synthase kinase 3 (P-GSK-3) [24].

Electron microscopy confirmed that the positive therapeutic effect of the mitochondria-targeted antioxidant was associated with the protection of mitochondria from structural damage. Moreover, animals receiving SkQR1 acquired a higher proliferative potential of kidney cells, compared to LPS-treated rats in the absence of SkQR1 injection. It is important to note that mitochondria-targeted antioxidants are a commonly used tool in the study of ROS production by mitochondria [43]. Consequently, we were able not only to demonstrate its therapeutic efficacy but, also, to confirm the pathological role of ROS produced by mitochondria using the antioxidant SkQR1. Our results are consistent with other studies which used SkQR1 as a therapeutic agent in models where oxidative stress was a major pathogenic cause of organ damage, such as ischemia/reperfusion of the kidney or brain [33,34], pyelonephritis [44], and toxic kidney damage [45]. Nephroprotective effects have also been demonstrated for other mitochondria-targeted antioxidants consisting of the lipophilic triphenylphosphonium cation (TPP^+^) attached to the ubiquinone antioxidant moiety (MitoQ) or plastoquinone (SkQ1) [41,46], including pathologies caused by endotoxin or bacterial sepsis [44,47,48,49]. However, earlier studies in this area were performed only in adult animals.

It should be noted that mitochondria-targeted antioxidants protect a wide range of organs whose dysfunction are caused by PAMPs. As the vascular endothelium is the primary target the endotoxin encounters after administration, likely causing its damage, this may be one main potential cause of multiple organ failure [50]. In this study, we also observed mitochondrial damage in renal endothelial cells of sepsis-exposed animals. Therefore, the renal endothelium, in addition to renal tubular cells, may present itself as a therapeutic target for mitochondrial antioxidants. Earlier, using electron microscopy, we revealed enhanced preservation of the mitochondria in endothelial cells from renal tissue in animals treated with SkQR1 after exposure to renal ischemia [51] and, recently, SkQR1 protection of the endothelium was demonstrated in vitro, in a model of tumor necrosis factor (TNF)-induced endothelial permeability [52].

The model of sepsis-induced AKI used in this study was closely relevant to the clinical situation associated with the congenital sepsis of newborns. From the results obtained, it can be concluded that even the prompt administration of antibiotics cannot completely prevent PAMP-associated damage to the renal tissue, since pathological cascades induced by systemic inflammation and the development of oxidative stress occur differently in a spatio-temporal manner. Under these circumstances, the mitochondria-targeted antioxidant SkQR1 can be used in newborns, in conjunction with conventional therapy, for the treatment of endotoxin-induced complications, such as AKI. Moreover, SkQR1 can counteract the reduced proliferative potential of renal tissue during AKI, thus enhancing the regenerative ability of the organ, which is otherwise diminished by the endotoxin.

As to the potential clinical application of mitochondria-targeted antioxidants to ameliorate the consequences of sepsis in infants, it should be kept in mind that there are clinical cases when it will be permissive to use mitochondria-targeted antioxidants for therapy in the pre-treatment mode. In some cases, predictors of early-onset neonatal sepsis have been found [53], for example, the presence of meconium in amniotic fluid is associated with a much higher probability of sepsis in the newborn. Thus, the risk group can be predicted in advance—prior to the manifestation of clinical signs—and, at this point, the use of mitochondria-targeted antioxidants will be beneficial.

## 5. Conclusions

In summary, the consequences of mitochondrial damage during neonatal sepsis-induced AKI indicates a necessity for the application of mitochondria-targeted antioxidants (in addition to conventionally used drugs) to cause neutralization/destruction of the pathogenic agent and, thus, afford protection of the mitochondria and prevent the development of pathological oxidative stress.

## Supplementary Materials

The following are available online at https://www.mdpi.com/2076-3921/8/6/176/s1.
