# Supplementary figures and images for: Mitochondrial Damage and Mitochondria-Targeted Antioxidant Protection in LPS-Induced Acute Kidney Injury
Source: Antioxidants (Basel). 2019 Jun 14;8(6):176. doi: 10.3390/antiox8060176 (PMC6617298; doi:10.3390/antiox8060176)

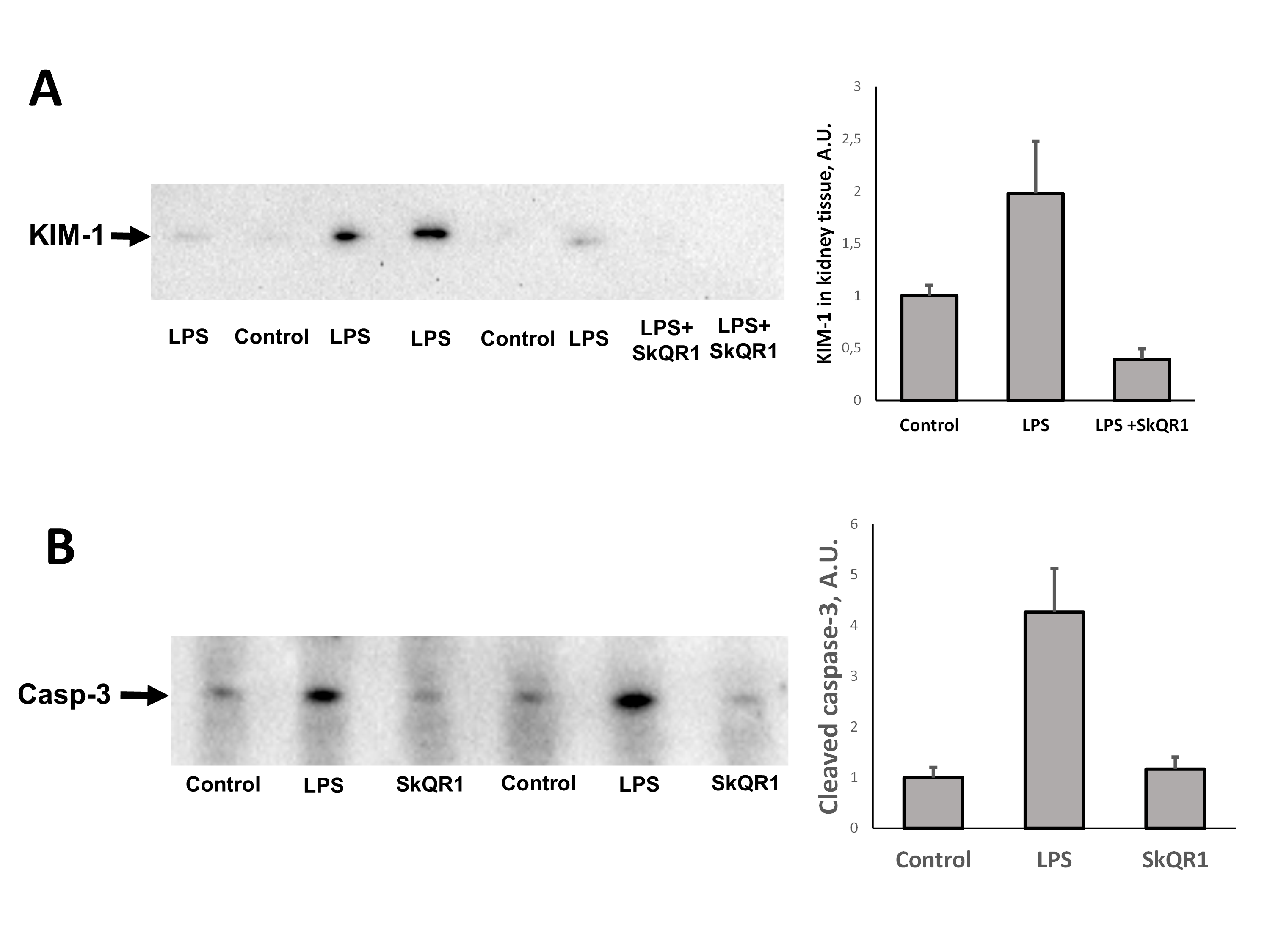

Supplement: Supplementary file 1 [file antioxidants-08-00176-s001.tif]
